# *In vitro*, *in vivo* and clinical studies comparing the efficacy of ceftazidime-avibactam monotherapy with ceftazidime-avibactam-containing combination regimens against carbapenem-resistant *Enterobacterales* and multidrug-resistant *Pseudomonas aeruginosa* isolates or infections: a scoping review

**DOI:** 10.3389/fmed.2023.1249030

**Published:** 2023-09-04

**Authors:** Abdullah Tarık Aslan, Yukiko Ezure, Juan Pablo Horcajada, Patrick N. A. Harris, David L. Paterson

**Affiliations:** ^1^Faculty of Medicine, UQ Centre for Clinical Research, University of Queensland, Brisbane, QLD, Australia; ^2^Infectious Diseases Department, Hospital del Mar, Institut Hospital Del Mar d’Investigacions Mèdiques (IMIM), Universitat Pompeu Fabra (UPF), Barcelona, Spain; ^3^CIBERINFEC, ISCIII – CIBER de Enfermedades Infecciosas, Instituto de Salud Carlos III, Madrid, Spain; ^4^Central Microbiology, Pathology Queensland, Royal Brisbane and Women’s Hospital, Brisbane, QLD, Australia; ^5^ADVANCE-ID, Saw Swee Hock School of Public Health, National University of Singapore, Singapore, Singapore

**Keywords:** antimicrobial resistance, carbapenem resistance, synergy, combination, monotherapy

## Abstract

**Introduction:**

Carbapenem-resistant *Enterobacterales* (CRE) and multidrug-resistant Pseudomonas aeruginosa (MDR-PA) infections are associated with a high risk of morbidity, mortality, and treatment costs. We aimed to evaluate *in vitro*, *in vivo* and clinical studies comparing the efficacy of ceftazidime-avibactam (CZA) combination regimens with CZA alone against CRE and/or MDR-PA isolates or infections.

**Methods:**

We systematically reviewed the relevant literature in CINAHL/MEDLINE, Pubmed, Cochrane, Web of Science, Embase, and Scopus until December 1, 2022. Review articles, grey literature, abstracts, comments, editorials, non-peer reviewed articles, non-English articles, and in vitro synergy studies conducted on single isolates were excluded.

**Results:**

22 *in vitro*, 7 *in vivo* and 20 clinical studies were evaluated. *In vitro* studies showed reliable synergy between CZA and aztreonam against metallo-β-lactamase (MBL)-producing isolates. Some studies indicated good in vitro synergy between CZA and amikacin, meropenem, fosfomycin and polymyxins against CRE isolates. For MDR-PA isolates, there are comparatively fewer *in vitro* or *in vivo* studies. In observational clinical studies, mortality, clinical cure, adverse events, and development of CZA resistance after exposure were generally similar in monotherapy and combination therapy groups. However, antibiotic-related nephrotoxicity and infection relapses were higher in patients receiving CZA combination therapies.

**Discussion:**

The benefit, if any, of CZA combination regimens in MDR-PA infections is elusive, as very few clinical studies have included these infections. There is no currently documented clinical benefit for the use of CZA combination regimens rather than CZA monotherapy. CZA combined with aztreonam for serious infections due to MBL producers should be evaluated by randomized controlled trials.

**Systematic review registration:**

https://www.crd.york.ac.uk/prospero/display_record.php?RecordID=278552, CRD42021278552.

## 1. Introduction

Antimicrobial resistance (AMR) is a global public health threat from which no country or region is spared (1–3). In a recent report, deaths attributable to bacterial AMR were estimated to be 1.27 million (95% UI 0.91–1.71) worldwide in 2019 (4). Each of the 6 bacteria mainly causing AMR-related mortality was responsible for more than 250,000 deaths: *Escherichia coli*, *Staphylococcus aureus*, *Klebsiella pneumoniae*, *Streptococcus pneumoniae*, *Acinetobacter baumannii*, and *Pseudomonas aeruginosa* by order of death count (4).

According to the Centre for Disease Control and Prevention (CDC) definition, *Enterobacterales* that are either resistant to any carbapenem or express a carbapenemase enzyme are defined as CRE (5). CRE are made up of a heterogeneous group of bacteria with various carbapenem resistance mechanisms, broadly classified as carbapenemase producers and non-carbapenemase producers. When treating infections caused by CRE, knowing whether the causative microorganism produces a carbapenemase enzyme and, if so, the type of carbapenemase expressed is very important to inform optimal treatment decisions. MDR-PA is defined as *P. aeruginosa* not susceptible to at least 1 of 3 antibiotic classes for which anti-pseudomonal activity is typically anticipated (6). MDR-PA generally arises as a consequence of a coaction of multiple resistance mechanisms (7, 8). Although carbapenemase expression is not a common resistance mechanism for *P. aeruginosa*, it can be detected frequently in some parts of the world (9, 10). Recently, a new phenomenon, difficult-to-treat resistant (DTR) *P. aeruginosa* was defined as *P. aeruginosa* isolates displaying non-susceptibility to all of the following antibiotics: piperacillin-tazobactam, aztreonam, cephalosporins, carbapenems, ciprofloxacin, and levofloxacin (11).

Ceftazidime-avibactam (CZA) is the first new generation β-lactam/β-lactamase inhibitor combination to come to market and consists of a third-generation cephalosporin and a new generation non-β-lactam/β-lactamase inhibitor (12). CZA has potent activity against KPC and OXA-48-like carbapenemases, extended-spectrum β-lactamases (ESBL) and AmpC β-lactamases, as well as against non-carbapenemase-producing CRE (13). CZA also has enhanced activity against MDR-PA strains with susceptibility rates ranging from 67% to 88% (13). Conversely, the combination of ceftazidime with avibactam does not confer antibacterial activity against carbapenem-resistant *A. baumannii* strains and metallo-β-lactamase-producing gram-negative bacteria (14). In observational studies, CZA-containing regimens have been shown to be associated with favorable clinical outcomes and a lower risk of nephrotoxicity compared to colistin-based regimens (15, 16). However, little is known on the benefits and harms of CZA combination therapies over CZA monotherapy.

The role of combination regimens in the treatment of infections with CRE and MDR-PA is a matter of long-standing debate (17, 18). The main expectation of clinicians from CZA combination regimens is to increase the clinical efficacy of CZA through synergistic interaction and to prevent the development of resistance to CZA (13). The latter is particularly critical, because several studies have demonstrated the emergence of resistance during or following therapy in 3.7%–8.1% of CZA receiving patients infected by CRE (19, 20). However, combination therapies may increase the likelihood of side effects and treatment costs.

In this scoping review, we aim to evaluate *in vitro*, *in vivo* and clinical studies comparing CZA monotherapy and CZA combination therapies. The insights generated by this scoping review can contribute to identifying the benefits and harms of CZA combination therapies over CZA monotherapy for infections caused by CRE and MDR-PA, and lead to hypotheses that can be tested in randomized clinical trials (RCTs).

## 2. Methods

This scoping review was performed in accordance with the Preferred Reporting Items for Systematic reviews and Meta-Analyses extension for Scoping Reviews (PRISMA-ScR) (21), Prospero ID: CRD42021278552.

### 2.1. Search strategy

We searched relevant literature in PubMed, MEDLINE, CINAHL, Cochrane, Embase, Scopus, and Web of Science in December 2022. The search terms, MeSH terms and publication filters used are presented in the Supplementary material. The references were imported into an EndNote (Clarivate Analytics, Philadelphia, Pennsylvania, United States) database, and duplicated and non-English studies were not included. References within the recruited articles were searched manually to capture additional studies.

### 2.2. Study selection

The titles and abstracts of all records were screened by 2 reviewers (AA and YE) independently to evaluate full-text eligibility. Any disagreements were resolved by discussion. Articles were included after full-text review if they met the following inclusion criteria: (1) original research, (2) *in vitro*, *in vivo* or clinical studies comparing CZA alone vs. CZA-containing combinations against CRE and/or MDR-PA isolates (regardless of their resistance mechanisms) or infections, and (3) published up to December 1, 2022. Otherwise, articles were excluded for the following reasons: (1) review articles; (2) the full text was not available; (3) grey literature; (4) abstracts, comments, letters, and editorials; (5) non-peer reviewed articles; (6) studies published in a language other than English; (7) *in vitro* synergy studies conducted on only one isolate.

### 2.3. Data extraction

Data were extracted into predesigned spreadsheets in duplicate by two independent reviewers. In *in vitro* studies, data pertaining with authors, publication year, country, AMR phenotype, predominant AMR mechanisms, CZA susceptibility rate, type of bacterial strain, number of isolates tested, method(s) used for antimicrobial susceptibility tests and criteria used for assessment of their results, type of *in vitro* synergy test applied, companion antibiotic(s) used, definition of synergism, additive effect, and antagonism, concentrations of CZA and companion antibiotics used in experiments, and number of repeats in experiments were extracted. In *in vivo* studies, authors, publication year, country, type of bacterial strain, AMR phenotype, main molecular determinants of AMR, type of *in vivo* infection model, number of isolates tested, method(s) used for antimicrobial susceptibility tests and criteria used for interpretation of their results, doses of CZA and companion antibiotics adjusted in experiments, and killing activity of tested antibiotics were extracted. In clinical studies, we extracted authors, publication year, country, the study design, statistical method, and relevant data, including the number of patients in comparison groups, type of companion antibiotics, *in vitro* activity of concomitant antibiotics, median time from onset of infection to active treatment, demographic information of patients, the infection type, number of bacteremic patients, carbapenem non-susceptibility rate of the causative pathogens, main AMR mechanism, presence of septic shock, and outcomes parameters of individual studies (i.e., all-cause mortality, clinical cure, microbiological cure, adverse events, acute kidney injury, emergence of CZA resistance and infection relapse). Because of the substantial heterogeneity of study designs, treatment regimens, definitions of study outcomes and combination regimens (*i.e.*, *in vitro* inactive antibiotics being considered as co-antibiotics in some studies), we did not perform quantitative synthesis of the data using a meta-analysis and a narrative synthesis was undertaken to evaluate the efficacy and safety of CZA monotherapy and CZA combination therapies.

## 3. Results

**Figure 1:**
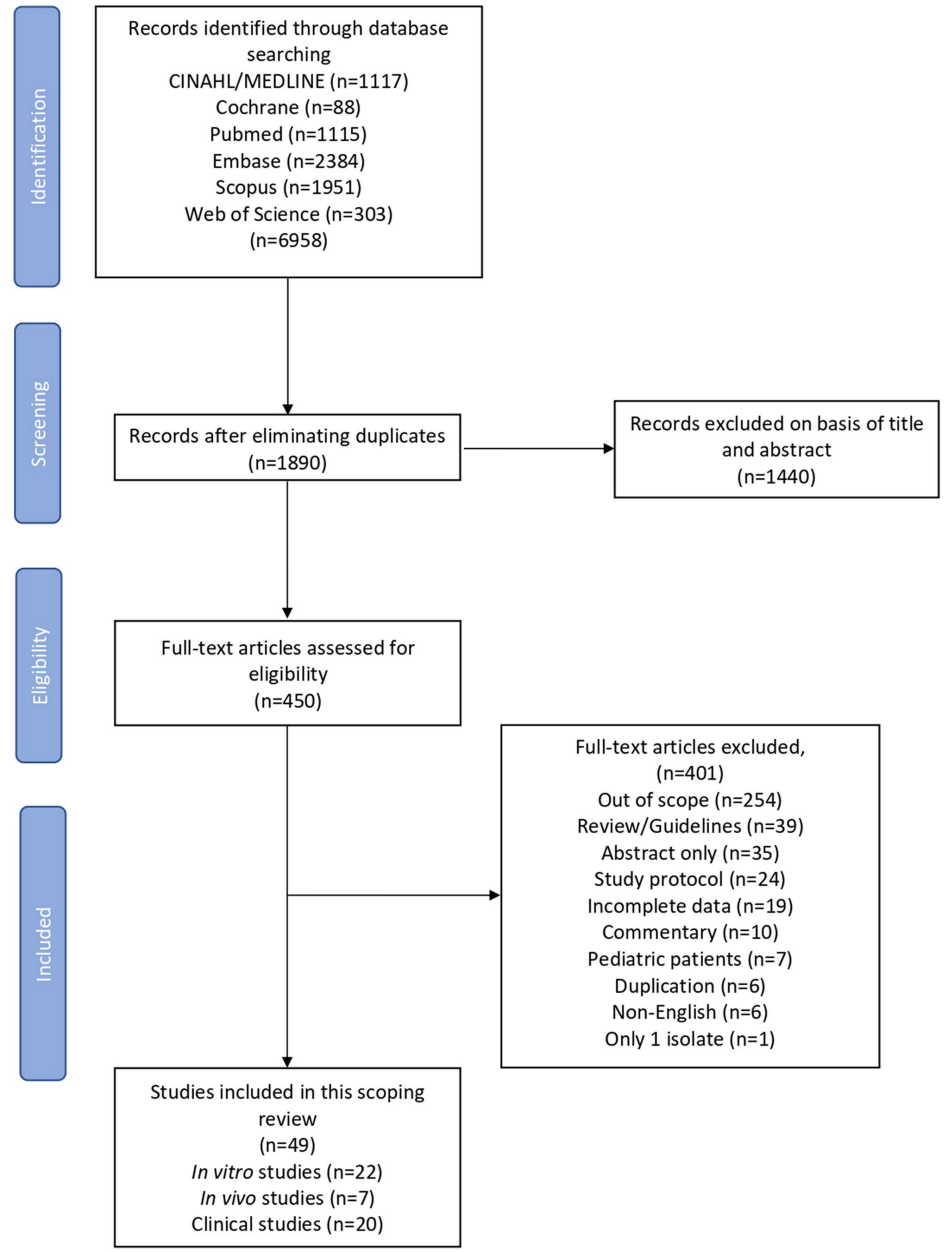
Flow chart of the inclusion and exclusion process.

In our database search, 6,958 studies were identified and 5,068 were excluded based on duplications. After reviewing titles and abstracts, 1,440 were not included and 401 articles were excluded for the reasons depicted in Figure 1. Overall, 49 studies comprising 22 *in vitro*, 7 *in vivo* and 20 clinical studies were included in the final data synthesis.

### 3.1. *In-vitro* studies

Some antibiotics can be combined with CZA to expand or enhance its antibacterial capacity. For example, avibactam protects aztreonam from the hydrolytic activity of ESBLs and AmpC β-lactamases, which are frequently co-produced by metallo-β-lactamase-producing gram-negative bacteria (22). As aztreonam is not hydrolyzed by metallo-β-lactamases, it can show significant antibacterial activity in the presence of CZA against metallo-β-lactamase-producing gram-negative bacteria (22). Furthermore, CZA plus aztreonam may substantially influence the “divisome” of these bacteria by acting on different types of penicillin-binding proteins (22). Another potential way of enhancing CZA’s *in vitro* activity is to take advantage of enhanced susceptibility to antibiotics brought about by mutations that compromise CZA’s activity. A unique mutation (D179Y) within or proximal to KPC-3-Ω-loop is responsible for CZA resistance in KPC-producing CRE isolates (19). Intriguingly, the same mutation may restore meropenem susceptibility in some strains. However, the susceptibility of these mutant strains to meropenem is generally unsustainable and, as a result, the clinical relevance of this finding is unclear (23). Nevertheless, the combination of CZA plus meropenem may open a new avenue for the treatment of KPC-producing CRE infections. Other combination regimens containing antibiotics with mechanisms of action different from CZA may increase cumulative antibacterial activity through synergistic interactions by less clearly explained mechanisms.

Among *in vitro* studies, 22 were included in final data synthesis (Figure 1). The main features of all included studies are shown in Table 1. Among 22 studies, 5 reported data on *Enterobacterales*, 10 *K. pneumoniae*, 3 *P. aeruginosa*, 2 both *K. pneumoniae* and *P. aeruginosa*, and 1 both *Enterobacterales* and *P. aeruginosa* and resistance mechanisms of tested pathogens were quite heterogeneous (Table 2). *In vitro* synergy was examined by time-kill (*n* = 13), gradient diffusion (*n* = 3), E-test MIC:MIC (*n* = 4), checkerboard (*n* = 4), and pharmacokinetic/pharmacodynamic (*n* = 2) studies. In four of these studies, two different methods were used to analyze the *in vitro* interactions of antibiotic combinations (29, 31, 32, 35). In studies utilizing E-test MIC:MIC, gradient diffusion and checkerboard, the fractional inhibitory concentration index (FICI) was calculated to define synergy. Synergy was defined as FICI ≤ 0.50, additivity >0.50 to ≤1.00, indifference >1.00 to ≤4.00, and antagonism >4.00. In time-kill and pharmacokinetic/pharmacodynamic studies, reduction in bacterial load ≥2log_10_ colony forming unit (CFU) as compared to most active single antibiotic was defined as synergy, while ≥2-log_10_ increase in bacterial burden was defined as antagonism. In approximately half of the studies (*n* = 10), the number of repetitions of the experiments was not provided (26, 29–32, 36, 40, 43–45). The remainder repeated the experiments either 2 (24, 25, 33, 35, 37–39, 41) or ≥ 3 (27, 28, 34, 42) times. All *in vitro* synergy studies were between CZA and commercially available antibiotics, except for one study in which Idowu et al. analyzed the synergy between CZA and tobramycin-cyclam conjugate, which abrogates the antimicrobial effects of tobramycin but potentiates the full antimicrobial activity of the concomitant antibiotic (32).

**Table 1:** Main characteristics of *in vitro* studies comparing ceftazidime-avibactam alone vs. ceftazidime-avibactam containing combinations.

Author	Year	Country	Bacteria	AMR phenotype	CZA susceptibility (%)	Number of isolates	AST method	AST criteria	Assay
Almarzoky Abuhussain et al. (24)	2018	United States	PA and KP	CR	100	6	BMD	CLSI	72-h PD Chemostat
Avery et al. (25)	2018	United States	*Enterobacterales*	CR	0	10	Gradient test and BMD	CLSI	Gradient diffusion
Avery et al. (26)	2019	United States	*Enterobacterales*	CR	NP	49	Gradient strip test	CLSI	Gradient diffusion
Biagi et al. (27)	2019	United States	*Enterobacterales*	CR	0	8	BMD	CLSI	Time-kill
Borjan et al. (28)	2020	United States	KP	CR	66.7	3	BMD	CLSI	Time-kill
Chen et al. (29)	2021	China	PA and *Enterobacterales*	CR	0	16	BMD	EUCAST and CLSI	Checkerboard and time-kill
Gaibani et al. (30)	2017	Italy	KP	CR	84.6	13	Gradient test	EUCAST	Gradient diffusion
Huang et al. (31)	2021	United States	KP	CR	100	4	BMD	CLSI	Time kill and hollow-fibre
Idowu et al. (32)	2019	Canada	PA	MDR or XDR	80	5	BMD	CLSI	Checkerboard and time-kill
Kara et al. (33)	2020	Turkey	*Enterobacterales*	CR	71	7	BMD	EUCAST	Time-kill
Lee et al. (34)	2021	United States	PA	CR	0	5	BMD	CLSI	Time-kill
Ma et al. (35)	2019	China	KP	CR	100	3	BMD	CLSI	Checkerboard and time-kill
Manning et al. (36)	2018	United States	KP	CR	100	10	Agar dilution	CLSI	Time-kill
Maraki et al. (37)	2021	Greece	KP	CR	0	40	E-test	CLSI	E-test MIC:MIC
Mikhail et al. (38)	2019	United States	PA and KP	MDR and CR	100	4	BMD	CLSI	Time-kill
Montero et al. (39)	2021	Spain	PA	XDR	66.7	21	BMD	CLSI	Time-kill
Nath et al. (40)	2018	United States	KP	CR	100	4	BMD	CLSI	Time-kill
Ojdana et al. (41)	2019	Poland	KP	CR	47	19	E-test	EUCAST	E-test MIC:MIC
Okoliegbe et al. (42)	2021	UK	PA	MDR	53	721	E-test	EUCAST	E-test MIC:MIC
Pragasam et al. (43)	2019	India	KP	CR	25	12	BMD	CLSI	Checkerboard
Romanelli et al. (44)	2020	Italy	KP	CR	100	10	E-test	EUCAST	E-test MIC:MIC
Shields et al. (45)	2018	United States	*Enterobacterales*	CR	100	24	BMD	CLSI	Time-kill

PA, *Pseudomonas aeruginosa*; KP, *Klebsiella pneumoniae*; AMR, antimicrobial resistance; CR, carbapenem-resistant; MDR, multidrug-resistant; XDR, extensively drug-resistant; CZA, ceftazidime-avibactam; AST, antimicrobial susceptibility testing; NP, not provided; BMD, broth microdilution; CLSI, Clinical and Laboratory Standard Institute; EUCAST, European Committee of Antimicrobial Susceptibility Testing; PD, Pharmacodynamic.

**Table 2:** *In vitro* synergy and antagonism of antibiotic combinations against carbapenem-resistant *Enterobacterales* and multidrug-resistant *Pseudomonas aeruginosa.*

Author	Bacteria	Main AMR mechanism	Number of isolates	Assay	Companion antibiotic(s)	Synergy rate (%)	Bactericidal synergy rate (%)	Antagonism rate (%)
Almarzoky Abuhussain et al. (24)	PA	NP	3	72-h PD Chemostat	Inhaled AMK	33.3	33.3	0
Almarzoky Abuhussain et al. (24)	KP	KPC	3	72-h PD Chemostat	Inhaled AMK	66.6	33.3	0
Avery et al. (25)	*Enterobacterales*	MBL +/− OXA-48	10	Gradient diffusion	ATM	90	NA	0
Avery et al. (26)	*Enterobacterales*	KPC, MBL, OXA-48	49	Gradient diffusion	FOS	0	NA	0
Biagi et al. (27)	*Enterobacterales*	Serine β-lactamases + MBL carbapenemases	8	Time-kill	ATM	87.5	87.5	0
Borjan et al. (28)	KP	KPC	3	Time-kill	POLB	0	0	66.6
Chen et al. (29)	PA and *Enterobacterales*	Carbapenemases and non-carbapenemases	16	Checkerboard	AMK	56.3	NA	0
Chen et al. (29)	PA and *Enterobacterales*	Carbapenemases and non-carbapenemases	2	Time-kill	AMK	100	100	0
Gaibani et al. (30)	KP	KPC	13	Gradient diffusion	GEN, CIP, TGC,	0, 0, 8	NA	0, 0, 0
Gaibani et al. (30)	KP	KPC	13	Gradient diffusion	IMI and MER	100, 100	NA	0, 0, 0
Huang et al. (31)	KP	KPC	4	Time kill	AMK, GEN	75, 100	75, 100	0, 0
Huang et al. (31)	KP	KPC	2	Hollow-fibre	AMK, GEN	50, 50	50, 50	0, 0
Idowu et al. (32)	PA	NP	5	Checkerboard	Tobramycin-cylam	100	NA	0
Kara et al. (33)	*Enterobacterales*	OXA-48	7	Time-kill	COL, TGC, TOB, DOR	71.4, 42.9, 57.1, 57.1	NP	NP
Lee et al. (34)	PA	Serine β-lactamases + MBL carbapenemases	5	Time-kill	ATM	80	80	0
Ma et al. (35)	KP	KPC	3	Time-kill	POLB	100	100	0
Ma et al. (35)	KP	KPC	3	Checkerboard	POLB	66.6	NA	0
Manning et al. (36)	KP	KPC	10	Time-kill	AMK, POLB, TGC	10, 30, 0	10, 30, 0	0
Maraki et al. (37)	KP	MBL	40	E-test MIC:MIC	ATM	97.5	NA	0
Mikhail et al. (38)	KP	KPC	2	Time-kill	ATM, FOS, AMK, MER, COL	100, 100, 100, 50, 50	50, 100, 100, 50, 50	0, 0, 0, 0, 0
Mikhail et al. (38)	PA	Various mechanisms	2	Time-kill	ATM, FOS, AMK, MER, COL	100, 0, 100, 100, 50	100, 0, 100, 0, 0	0, 0, 0, 0, 0
Montero et al. (39)	CZA susceptible PA	Various mechanisms	14	Time-kill	AMK, ATM, MER, COL	57.1, 14.3, 7.1, 42.9	0, 7.1, 0, 14.3	0, 7.1, 35.7, 0
Montero et al. (39)	CZA resistant PA	MBL and class A carbapenemases	7	Time-kill	AMK, ATM, MER, COL	71.4, 57.1, 28.6, 85.7	42.9, 28.6, 28.6, 42.9	0, 14.3, 0, 0
Nath et al. (40)	KP	KPC	4	Time-kill	MER, POLB, AMK	50, 25, 50	25, 25, 50	0, 25, 0
Ojdana et al. (41)	KP	KPC, OXA-48, NDM	19	E-test MIC:MIC	ERT, FOS, TGC	47, 47, 5	NA	5, 0, 0
Okoliegbe et al. (42)	PA	NP	721	E-test MIC:MIC	TOB	30	NA	0
Pragasam et al. (43)	KP	NDM, OXA-48, NDM + OXA-48	12	Checkerboard	ATM	100	NA	0
Romanelli et al. (44)	KP	KPC	10	E-test MIC:MIC	MER, IMI, ERT, FOS	100, 100, 100, 0	NA	0, 0, 0, 0
Shields et al. (45)	*Enterobacterales*	KPC	24	Time-kill	COL	12.5	8.3	46

PA, *Pseudomonas aeruginosa*; KP, *Klebsiella pneumoniae*; CZA, ceftazidime-avibactam; MBL, metallo-β-lactamases; AMR, antimicrobial resistance; NP, not provided; AMK, amikacin; ATM, aztreonam; FOS, fosfomycin; POLB, polymyxin B; GEN, gentamicin; CIP, ciprofloxacin; TGC, tigecycline; IMI, imipenem; MER, meropenem; COL, colistin; ERT, ertapenem, TOB, tobramycin; NA, not applicable.

Table 2 presents the *in vitro* synergy and antagonism rates of the combination regimens tested for CRE and MDR-PA. The synergy rate between CZA and aztreonam was >80% in all studies using metallo-β-lactamases-producing CRE and MDR-PA (25, 27, 34, 37, 43). Even though the results were quite heterogeneous as shown in Table 2, CZA plus meropenem or imipenem (30, 38, 40, 44) and CZA plus amikacin can show synergistic activity, particularly against CRE isolates (24, 29, 31, 38, 39). Similarly, synergy rates for the combination of CZA plus fosfomycin were highly variable against CRE isolates (26, 38, 41, 44) but very low against MDR-PA in one study (38). In other combinations, the synergy rates were generally low against CRE and MDR-PA isolates (Table 2). For CZA-resistant MDR-PA isolates, Montero et al. found high rate of synergy between colistin and CZA (39). Conversely, Shields et al. demonstrated significant antagonism between colistin and CZA against KPC-expressing CZA-susceptible *Enterobacterales* isolates (45).

### 3.2. *In vivo* infection models

After assessment of relevant articles, seven were included in narrative data synthesis. All studies except one from Canada were reported from the United States. Five studies included carbapenem-resistant *Klebsiella pneumoniae* (CRKP) isolates (3 KPC, 1 NDM and 1 NDM plus OXA-48 producers) and two assessed MDR-PA. The *in vivo* infection models used are as follows: *Galleria mellonella* survival model (*n* = 3), neutropenic mouse thigh infection model (*n* = 2), and neutropenic mouse pneumonia model (*n* = 2).

In a *Galleria mellonella* survival model, CZA (1.56/1.56 mg/kg) ensured 70% survival of MDR-PA-challenged larvae after 24 h, while CZA (1.56/1.56 mg/kg) plus tobramycin-cyclam (3.12 mg/kg) provided 100% survival after 24 h (32). In another *Galleria mellonella* survival model, human simulated regimens of CZA had better antibacterial activity when combined with either meropenem or amikacin against KPC-producing *K. pneumoniae* isolates (*n* = 2) with CZA MIC level of 8/4 mg/L (40). In contrast, these combination regimens did not have significant killing effect compared to single CZA regimen against *K. pneumoniae* isolates (*n* = 2) with lower (1/4 mg/L) MIC of CZA in virtue of excellent *in vivo* activity of CZA monotherapy (40). In the last *Galleria mellonella* survival model, CZA and polymyxin B were tested with concentrations of being four times MIC alone and in combination against KPC-producing *K. pneumoniae* isolates (*n* = 3). The CZA plus polymyxin B combination did not significantly improve larval survival in comparison with CZA alone against any strain (28). Likewise, mean survival times were similar in both groups.

In two neutropenic mouse thigh infection models, the authors demonstrated significant synergy between CZA plus aztreonam and CZA plus fosfomycin, respectively. Marshall et al. showed that as compared to CZA (32 mg/kg q8h) and aztreonam (32 mg/kg q8h) alone, CZA (32/8 mg/kg q8h) plus aztreonam (32 mg/kg q8h) combination reduces bacterial titers of an NDM-producing *K. pneumoniae* by 3.79 log_10_ CFU and 2.08 log_10_ CFU per thigh, respectively (22). Papp-Wallace et al. revealed that the human simulated doses of CZA and fosfomycin combination provides significant reduction in CFUs of CZA-susceptible MDR-PA isolates (*n* = 2) by approximately 5 logs, compared with the vehicle-treated control (46). Separate administration of CZA and fosfomycin reduced bacterial loads by approximately 1 log and 2 logs, respectively, compared with those of the vehicle-treated control (46).

Huang et al. performed a neutropenic mouse pneumonia model with inoculation of a KPC-producing *K. pneumoniae* isolate (31). At 24 h, mean bacterial lung concentrations (± the standard deviation) for the untreated control group, gentamicin, CZA, and gentamicin plus CZA combination were 8.97 ± 0.09, 4.64 ± 0.34, 5.95 ± 0.25, and 2.95 ± 1.07 log_10_ CFU/lung, respectively. In a neutropenic murine lung infection model utilizing NDM, OXA-48 and CTX-M expressing *K. pneumoniae* strain (*n* = 1), the humanized dose of CZA monotherapy (2.5 g q8h) led to substantial *in vivo* killing activity (2.43 log_10_ CFU reduction at 24 h), and consequently, no significant benefit was found compared to CZA monotherapy with CZA plus aztreonam (2 g every 6 h) or CZA plus tigecycline (100 mg q12h) (2.54 log_10_ CFU reduction with CZA plus aztreonam and 2.15 log_10_ CFU reduction with CZA plus tigecycline at 24 h) (47). Even with CZA plus aztreonam plus tigecycline combination, bacterial load can be reduced by 2.18 log_10_ CFU at 24 h, which did not provide significant advantage over CZA monotherapy (47).

In summary, CZA plus fosfomycin and CZA plus aztreonam were shown to have more robust antibacterial activity than CZA alone in neutropenic mouse thigh infection models utilizing MDR-PA and NDM-producing *K. pneumoniae*, respectively. Intriguingly, in a neutropenic murine lung infection model, CZA alone showed excellent killing activity against NDM, OXA-48 and CTX-M-expressing *K. pneumoniae*, raising the clinical impact of NDM production on these infections into question (47). In another study, more effective antibacterial activity was shown with CZA plus meropenem or amikacin against KPC-producing *K. pneumoniae* strains if CZA MIC level is near to susceptibility breakpoint. For other combinations, no benefit of the combination regimens was demonstrated over CZA alone.

### 3.3. Clinical studies

Of the 20 studies evaluated, 9 were retrospective multi-center cohort (48–56), 8 retrospective single-center cohort (15, 57–63), 1 prospective multi-center cohort (64), 1 prospective single-center cohort (65) and 1 multi-center case–control study (66). There were no RCTs which address combination therapy and monotherapy with CZA. The number of patients included ranged from 13 to 577. Only four studies assessed bloodstream infections alone and the rest included patients with different types of infections. These studies were conducted in various countries as follows: Spain (6), United States (4), Italy (4), Greece (1), Saudi Arabia (1), Brazil (1), India (1), China (1), Europe, Israel and Australia (1). As shown in Table 3, *in vitro* susceptibility of carbapenems was not tested in two studies (55, 60) and carbapenem non-susceptibility rate was <100% among isolates collected in four studies (49, 57, 64, 65). Similarly, CZA susceptibility rate was not documented in three studies (49, 50, 55), as well as it was found as 89.5% and 79% in two studies, respectively (57, 61). More than half of the studies had CRE infections, while six included CRKP infections (15, 52–54, 56, 60). MDR/XDR-PA infections were assessed only in one study (57) and two studies included mixed types of infections caused by CRE or carbapenem-resistant *P. aeruginosa* (CRPA) and CRE or MDR-PA, respectively (49, 51). The causative pathogens were susceptible to concomitantly administered antibiotic(s) in the vast majority of the studies, five studies did not document *in vitro* antimicrobial activity of partner antibiotics (48, 56, 58, 61, 66) and 70% of concomitant antibiotics had *in vitro* antimicrobial activity against the causative pathogens in one study (62). Intriguingly, none of the co-administered antibiotics were *in vitro* active against the identified pathogens in one study (53). Although median time between the onset of infection and administration at least one *in vitro* active antibiotic was not reported in 12 studies, it was generally quite long in other studies as shown in Table 3. Except four studies included only bloodstream infections, secondary bacteremia was frequent in other studies (Table 3). The definitions of investigated outcomes were highly heterogeneous and are portrayed in Table 4. In three studies, 30-day mortality rate was explored along with mortality rates at other time points (56, 63, 65). Since the majority of the studies included in this scoping review analyzed 30-day mortality, we preferred to report the 30-day mortality rates in these studies. All-cause mortality was investigated in all but one and ranged from 6.3% to 47.6% in patients treated with CZA monotherapy and from 0% to 44.0% in those receiving CZA combination regimens (Table 5). Only four studies performed a multivariable analysis while comparing monotherapy and combination therapies. In the study conducted by Zheng et al., CZA as a component of combination regimens was found to be associated with lower risk of mortality in comparison with CZA monotherapy (24.4% vs. 47.6%; HR, 0.167; 95 CI, 0.06–0.46) (53). In contrast, CZA combination regimens were associated with higher mortality in another study (27.8% vs. 11.8%; HR, 2.49; 95% CI, 1.10–5.63) (64). Alqahtani et al. reported no significant difference in mortality between CZA combination therapies and CZA monotherapy (27% vs. 16%; OR, 1.5; 95% CI, 0.61–3.64) (63). Lastly, in an Italian retrospective matched cohort study, there was no difference between CZA plus fosfomycin and CZA ± other antibiotic groups in terms of 30-day mortality (14.8% vs. 18.0%; HR, 0.72; 95% CI, 0.28–1.85) (56).

**Table 3:** Main characteristics of clinical studies comparing ceftazidime-avibactam vs. ceftazidime-avibactam-containing combination regimens.

Author	Country	Number of sites	Median time to active therapy	*In vitro* susceptibility rate of partner antibiotic (%)	Age^a^	Number of bacteremic patients (*n*, %)	Carbapenem non-susceptibility rate (%)	Main AMR mechanism	Presence of septic shock (*n*, %)	SOFA score^a^	APACHE II score^a^
Ackley et al. (48)	United States	18	25.0 (2.7–56.7) hours	NP	62 (51–69)	44 (41.9)	100	KPC	NP	NP	26 (22–30)
Balandín et al. (49)	Spain	11	NP	100	61.4 ± 14.0	22 (32.4)	84.2	OXA-48 and KPC	40 (58.8)	7.6 ± 4.0	19.5 ± 7.5
Corbella et al. (57)	Spain	1	6 (1–9) days	100	65.1 ± 15.9	9 (14.8)	98.4	NP	NP	NP	NP
De la Calle et al. (58)	Spain	1	NP	NP	58.8 ± 16.0	8 (33.3)	100	OXA-48	4 (16.7)	3.3 ± 2.8	NP
King et al. (50)	United States	9	8 (5–22) days	100	60 (51–69)	23 (38.3)	100	NP	NP	NP	NP
Shields et al. (59)	United States	1	NP	100	62 (19–91)	22 (28.6)	100	KPC	NP	5 (0–20)	NP
Shields et al. (15)	United States	1	NP	100	66 (32–91)	13 (100.0)	100	KPC	NP	NP	20 (16–33)
Sousa et al. (65)	Spain	1	5 (0–13) days	100	64 (26–86)	26 (45.6)	97.3	OXA-48	NP	NP	24 (8–45)
Temkin et al. (51)	Europe, Israel, Australia	15	NP	100	61 (47–67)	26 (68.4)	100	KPC and OXA-48	NP	NP	NP
Tumbarello et al. (52)	Italy	17	7 (3–9) days	100	60 (27–79)	104 (100.0)	100	KPC	34 (32.7)	NP	NP
Zheng et al. (53)	China	2	NP	0	60.9 ± 17.1	NP	100	NP	NP	NP	17.5 (14.8–20)
Tumbarello et al. (54)	Italy	22	NP	100	66 (56–76)	391 (67.8)	100	KPC	100 (17.3)	NP	NP
Castón et al. (60)	Spain	1	3 (1–5) days	100	70 (54–79)	24 (51.1)	NP	KPC	25 (53.2)	3 (2–6)	14 (9–19)
Rathish et al. (61)	India	1	NP	NP	53.2 ± 17.3	50 (48.5)	100	NP	30 (29.0)	4.3 ± 3.2	NP
Ianconne et al. (62)	Italy	1	NP	70	NP	23 (100.0)	100	KPC	NP	NP	NP
Guimarães et al. (66)	Brazil	3	NP	NP	50.5	12 (41.4)	100	KPC	NP	NP	NP
Castón et al. (55)	Spain	14	2 (1–4)	100	67 (56–77)	72 (38.1)	NP	OXA-48 and KPC	36 (19.0)	3 (1–6)	14 (9–19)
Karaiskos et al. (64)	Greece	14	NP	100	60.9 ± 17.1	95 (64.6)	99	KPC and OXA-48	50 (34.0)	6.7 ± 4.2	16.5 ± 7.6
Alqahtani et al. (63)	Saudi Arabia	1	1 day^b^	100	62 ± 19	54 (25.6)	100	OXA-48, NDM and OXA-48 + NDM	NP	NP	NP
Oliva et al. (56)	Italy	2	NP	NP	68 (57–78)	122 (100.0)	100	KPC	25 (20.5)	NP	NP

NP, not provided; AMR, antimicrobial resistance; SOFA, sequential organ failure assessment; APACHE II, Acute Physiology and Chronic Health Evaluation II.

a The results were presented as median (min-max) or mean (±standard deviation) depending on how they were given in the original articles.

b The range (min-max) of median time to active therapy is not provided in the original article.

**Table 4:** Definitions and time points used in clinical studies to define mortality, clinical cure, microbiological cure, and infection relapse.

Outcomes	Definitions or time points	References
Mortality	All-cause mortality by day-30	(49, 50, 52–57, 60, 63, 65)
	In-hospital all-cause mortality	(51, 56, 61, 62)
	All-cause mortality by day-90	(15, 48, 58)
	All-cause mortality by day-28	(64)
	All-cause mortality by day-14	(56, 66)
Clinical cure	Resolution of presenting symptoms and signs of the infection by the end of therapy	(49–51, 56, 61, 63)
	Resolution of fever and other signs and symptoms attributable to infection in the absence of relapse by day-14	(57)
	Resolution of fever and other signs and symptoms attributable to infection in the absence of relapse by day-30	(58)
	Resolution of signs and symptoms, survival, absence of relapse and absence of microbiological failure by day-30	(59)
	Resolution of signs and symptoms, survival and absence of relapse by day-30 and microbiological cure within 7 days	(15)
	Resolution of signs and symptoms within 7 days of treatment initiation	(65)
	Absence of death, infection relapse and signs and symptoms at day-14	(60)
	Discharge from the hospital	(62)
	Resolution of signs and symptoms when antibiotics are discontinued, survival by day-30, sterilization of blood culture within 7 days for bacteremic cases, absence of relapse by day-90	(48)
Microbiological cure	Eradication of microorganism at the end of therapy	(49–51)
	Negative blood culture 72 h after treatment onset	(56)
	Eradication of microorganism within 7 days of treatment	(56, 59)
	Eradication of the microorganism at the end of therapy and/or within 7 days of treatment	(65)
	Eradication of microorganism at 30-day	(53, 60)
Infection relapse	Presence of same microorganism at the same location within 90-days of the index infection	(48, 57)
	Presence of same microorganism at the same location during the follow-up	(49)
	Microbiological failure and concomitant signs of infection within 90 days	(65)
	Onset of a second microbiologically documented KPC-KP infection in a patient whose original infection had been classified as a clinical cure	(54, 56)
	Emergence of recurrent BSI during hospitalization	(62)
	Reinfection with the same organism within 30 days after completion of therapy	(63)

**Table 5:** Studies assessing clinical outcomes of ceftazidime-avibactam monotherapy vs. ceftazidime-avibactam-containing combination regimens.

Author	Year	Study design	Number of patients	Type of infection	Type of pathogen	Mortality rate (%)	Clinical cure rate (%)	Microbiological cure rate (%)	Adverse events rate (%)	AKI rate (%)	Emergence of CZA resistance rate (%)	Infection relapse rate (%)
Ackley et al. (48)	2020	R, MC, C	41 vs. 64	Mix	CRE	22.0 vs.31.2	63.4 vs. 60.9	NP	34.2 vs. 34.4	24.4 vs. 25.0	7.3 vs. 0	22.0 vs. 9.4
Balandín et al. (49)	2022	R, MC, C	34 vs. 34	Mix	CRE and MDR-PA	32.4 vs. 32.4	79.4 vs. 67.6	35.2 vs. 38.2	NP	NP	NP	0 vs. 0
Corbella et al. (57)	2022	R, SC, C	32 vs. 29	Mix	MDR/XDR-PA	6.3 vs. 20.7	62.5 vs. 44.8	NP	NP	0 vs. 3.4	0 vs. 0	6.5 vs. 20.0
De la Calle et al. (58)	2019	R, SC, C	14 vs. 10	Mix	CRE	14.3 vs. 30.0	64.3 vs. 60.0	NP	NP	NP	NP	NP
King et al. (50)	2017	R, MC, C	33 vs. 27	Mix	CRE	30.0 vs. 33.0	67.0 vs. 63.0	45.4 vs. 63.0	0 vs. 0	0 vs. 0	NP	NP
Shields et al. (59)	2018	R, SC, C	53 vs. 24	Mix	CRE	NP	56.6 vs. 50.0	67.9 vs. 66.6	NP	NP	11.3 vs. 8.3	NP
Shields et al. (15)	2017	R, SC, C	8 vs. 5	BSI	CRKP	12.5 vs. 0.0	75.0 vs. 100.0	NP	NP	14 vs. 25	NP	NP
Sousa et al. (65)	2018	P, SC, C	46 vs. 11	Mix	CRE	21.7 vs. 27.3	80.4 vs. 63.6	67.4 vs. 54.5	NP	2.2 vs. 9.1	0 vs. 0	8.7 vs. 18.2
Temkin et al. (51)	2017	R, MC, C	13 vs. 25	Mix	CRE and CRPA	26.7 vs. 44.0	61.5 vs. 72.0	61.5 vs. 64.0	NP	NP	0 vs. 0	NP
Tumbarello et al. (52)	2019	R,MC, C	22 vs. 82	BSI	CRKP	40.9 vs. 35.4	NP	NP	NP	NP	NP	NP
Zheng et al. (53)	2021	R, MC, C	21 vs. 41	Mix	CRKP	47.6 vs. 24.4	NP	42.9 vs. 61.0	NP	NP	NP	NP
Tumbarello et al. (54)	2021	R, MC, C	165 vs. 412	Mix	CRKP	26.1 vs. 25.0	NP	NP	3.0 vs. 3.6	NP	3.6 vs. 3.4	7.9 vs. 12.1
Castón et al. (60)	2020	R, SC, C	34 vs. 13	Mix	CRKP	26.5 vs. 15.4	55.9 vs. 69.2	64.7 vs. 46.2	NP	NP	11.8 vs. 15.4	NP
Rathish et al. (61)	2021	R, SC, C	69 vs. 34	Mix	CRE	29.0 vs. 23.5	71.0 vs. 76.5	NP	NP	NP	NP	NP
Ianconne et al. (62)	2020	R, SC, C	3 vs. 20	BSI	CRE	33.3 vs. 25.0	66.7 vs. 75.0	NP	NP	NP	0 vs. 10.0	0 vs. 20.0
Guimarães et al. (66)	2019	P, MC, CC	15 vs. 14	Mix	CRE	40.0 vs. 21.4	NP	NP	NP	NP	NP	NP
Castón et al. (55)	2022	R, MC, C	133 vs. 56	Mix	CRE	14.3 vs. 12.5	NP	NP	NP	NP	NP	NP
Karaiskos et al. (64)	2021	P, MC, C	68 vs. 79	Mix	CRE	11.8 vs. 27.8	NP	NP	NP	NP	1.5 vs. 1.3	NP
Alqahtani et al. (63)	2022	R, SC, C	119 vs. 92	Mix	CRE	16.0 vs. 27.1	86.6 vs. 67.4	NP	15.1 vs. 17.4	6.7 vs. 8.7	NP	7.6 vs. 13.0
Oliva et al. (56)	2022	R, MC, C	61 vs. 61	BSI	CRKP	14.8 vs. 18.0	75.4 vs. 60.7	76.7 vs. 94.6	NP	NP	0 vs. 3.3	11.5 vs. 26.2

R, retrospective; P, prospective; MC, multi-centre; SC, single-centre; C, cohort; CC, case–control; BSI, bloodstream infection; CRE, carbapenem-resistant Enterobacterales; multidrug-resistant *Pseudomonas aeruginosa*; XDR-PA, extensively drug-resistant *Pseudomonas aeruginosa*; CRKP, carbapenem-resistant *Klebsiella pneumoniae*; NP, not provided; CZA, ceftazidime-avibactam.

Among all studies, 14 assessed clinical cure or improvement of recruited patients by applying definitions shown in Table 4. The clinical cure rate ranged from 55.9% to 86.6% in the CZA monotherapy group and 44.8% to 100% in the CZA combination therapy group. Only the study showing lowest rate of clinical cure with CZA combination regimens executed multivariable analysis and reported that CZA combination therapy is significantly associated with lower clinical cure rate as compared to CZA monotherapy (44.8% vs. 62.5%; OR, 0.02; 95% CI, 0.01–0.38) (57). Only eight studies documented microbiological eradication rates in the CZA monotherapy and combination therapy groups (Table 5). While they were between 35.2 and 67.9% in the monotherapy groups, they ranged from 38.2% to 66.6% in the CZA combination therapy groups. In the study conducted by Oliva et al., blood culture negativity 72 h after treatment initiation for KPC-producing CRKP was higher in CZA plus fosfomycin arm than in CZA ± another antibiotic arm. However, there was no difference between the microbiological eradication rates of the two groups on the 7th and 14th days (56). Adverse event rates of monotherapy and combination therapies were reported in four studies and they seem to be quite similar between the two treatment groups in all of these studies (Table 5). Additionally, six studies explored the acute kidney injury (AKI) rates of CZA monotherapy and CZA combination regimens (Table 5). The AKI rates were numerically higher in CZA combination regimens in all but one study in which no AKI episode was detected in both groups (50).

One of the biggest issues of CZA treatment is the development of resistance during or following treatment. Except one study conducted by Ackley et al. (48), none of nine studies demonstrated a substantial difference between monotherapy and combination therapies in terms of development of CZA resistance during or following therapy (Table 5). In the study of Ackley et al., resistance development was not observed in any patients treated with CZA combination regimens, while 7.3% of monotherapy group developed resistance against CZA (48). Similarly, except the studies conducted by Ackley et al. and Oliva et al. (48, 56), five studies indicated numerically higher infection relapse rates in CZA combination therapy groups and no infection relapse was observed in one study (Table 5).

## 4. Discussion

This scoping review systematically documents the existing data on *in vitro*, *in vivo* and clinical studies comparing CZA monotherapy and CZA combination therapies. *In vitro* studies elucidated reliable synergy between CZA and aztreonam against metallo-β-lactamase-producing CRE and MDR-PA isolates. Consistently, in a recent prospective cohort study including 102 patients with bloodstream infection caused by metallo-β-lactamase-producing CRE being treated with either CZA plus aztreonam (*n* = 52) or colistin and tigecycline-based regimens (*n* = 50), propensity-score-adjusted multivariable analysis showed a significant association between CZA plus aztreonam and lower 30-day mortality (HR 0.37, 95% CI 0.13–0.74) (67). It should be underlined that absence of data from RCTs, absence of standardized antimicrobial susceptibility testing method and clinical interpretative breakpoints approved for this combination and lack of availability of aztreonam in some countries are important barriers to routine use of this combination regimen in daily practice. Furthermore, aztreonam (8 g daily) can lead to elevations in hepatic transaminases (68).

In general, *in vitro* studies showed reliable synergy between CZA and meropenem against KPC-producing CRE isolates. Considering the restoration of meropenem susceptibility in some CRE isolates carrying KPC-Ω-loop mutations and results of *in vitro* synergy studies, CZA plus meropenem can be considered to test in clinical studies as an alternative combination regimen for treatment of KPC-producing CRE infections.

CZA plus amikacin is one of the most common combination regimens investigated in *in vitro* synergy studies. The great majority of these studies found good synergy between amikacin and CZA against CRE isolates as well as a low number of studies showed good synergy between the two antibiotics against MDR-PA strains (24, 29, 31, 38, 39). However, two studies demonstrated low rate of *in vitro* synergy between the two antibiotics (36, 40). These conflicting findings are likely stemmed from differences in methods used to test *in vitro* synergy, doses of antibiotics adjusted, molecular backgrounds of tested microorganisms, and MIC levels of CZA and amikacin against pathogens used in these experiments.

The synergy between CZA and polymyxins was frequently explored in *in vitro* studies. While some studies reported high rate of synergy between these two antibiotics (33, 35, 39), others showed very low synergy between polymyxins and CZA (28, 36, 40, 45). Two studies also underlined the high risk of antagonism between polymyxins and CZA against KPC-producing *K. pneumoniae* isolates (28, 45). For other combinations, existing studies generally demonstrated low synergy between CZA and these antibiotics.

As CZA generally has an excellent *in vitro* antibacterial activity against CRE and MDR-PA isolates, synergistic interactions would be more useful for isolates with CZA MICs very close to the susceptibility breakpoints or non-metallo-β-lactamase-producing CZA-resistant isolates (69–72). Only two studies investigated *in vitro* synergy between CZA and partner antibiotics against non-metallo-β-lactamase-producing MDR-PA and/or CRE isolates with <50% CZA susceptibility rate and showed favorable results between CZA and amikacin (29, 37). Likewise, Nath et al. showed that there is no need to combine CZA with meropenem for highly CZA-susceptible KPC-producing *K. pneumoniae* isolates. In contrast, CZA plus meropenem yielded positive results for these isolates with a higher CZA MIC level (8/4 mg/L) (40). Although one study demonstrated enhanced synergy between CZA and colistin in CZA-resistant MDR-PA isolates, more studies underpinning this finding are needed (39).

There are very small numbers of *in vivo* studies comparing antibacterial activities of CZA combination regimens vs. CZA monotherapy. These studies were typically conducted on very few pathogens using various types of *in vivo* infection models. Therefore, it is not possible to confirm the activity of a specific combination regimen with at least 2 different studies performed using the same *in vivo* infection model testing the same bacterial species with a similar CZA MIC level and molecular resistance profile. As a result, it is highly unlikely to draw a firm conclusion from these *in vivo* studies’ results.

Even though *in vitro* and *in vivo* studies indicated that some combination regimens have better antimicrobial activity than CZA alone against CRE and MDR-PA, clinical studies comparing the clinical efficacy and safety of these combination regimens compared to CZA monotherapy play a key role in determining optimal therapeutic regimens for patients infected with these pathogens (73, 74). Evidence on CZA monotherapy and combination therapies comes from observational and case–control studies involving patients with mixed-type infections of varying clinical severity and these studies have significant limitations that should be highlighted. First, CZA combination regimens are basically composed of two or more antibiotics with variable *in vitro* antimicrobial activity. Although great majority of studies defined combination therapy as CZA plus at least one *in vitro* active antibiotic, in two studies, overall *in vitro* susceptibility rates of partner antibiotics used were 0% and 70%, respectively (53, 62). More importantly, individual data for specific combination regimens (e.g., CZA plus amikacin) were not presented in the vast majority of studies. Second, these studies typically include infections due to pathogens with various patterns of antimicrobial resistance, ranging from strains susceptible to carbapenems to those resistant to all available antimicrobials. Of these studies, little explored clinical outcomes of non-carbapenemase producers, CRE other than *K. pneumoniae* and MDR-PA. Furthermore, the definitions of combination therapy, clinical cure/improvement and infections relapses as well as mortality time points (e.g., 30-day, 90-day, in-hospital) were highly variable among the studies. Finally, multivariable analyses were seldom performed in these studies mainly due to the small number of patients included. Overall, in clinical studies published up to now, mortality, clinical cure, adverse events, and development of CZA resistance after exposure were generally similar in CZA monotherapy and combination therapy groups. However, antibiotic-related acute kidney injury and infection relapses were numerically higher in patients receiving CZA combination therapies. As only one study compared CZA monotherapy vs. CZA combination therapies against MDR/XDR-PA infections, more data are urgently needed to appreciate the differences between the two therapies against MDR-PA infections (57).

In tandem with our findings, recent European Society of Clinical Microbiology and Infectious Diseases (ESCMID) guidelines and Infectious Diseases Society of America (IDSA) guidance document recommend not using CZA as a component of combination regimens for CRE infections except for those caused by metallo-β-lactamase producers where CZA can be combined with aztreonam (75, 76). ESCMID guidelines do not also make any firm suggestion on the use of CZA for CRPA infections even as a monotherapy due to shortage of adequate evidence. In contrast, the IDSA guidance document does recommend CZA monotherapy for severe DTR-PA infections, but does not recommend prescribing CZA combination regimens for these infections (76). Considering the paucity of clinical data to make a recommendation on treatment decisions of CRE and MDR-PA infections, RCTs comparing CZA monotherapy vs. combination therapies for infections caused by these resistant pathogens (i.e., pathogen-directed RCTs) are needed. Given the paucity of clinical and preclinical data that consistently shows that a particular CZA-containing combination regimen(s) may be beneficial against CRE and/or MDR-PA infections, it is extremely difficult to prioritize any combination regimen in a pathogen-directed RCT.

Currently, treatment approaches for CRE and MDR-PA infections are constantly evolving, and precision medicine has recently gained significant popularity. In this innovative multistep medicinal approach, patient management is individualized based on patient and pathogen characteristics (13). Similarly, aminoglycoside-containing regimens should be individualized based on aminoglycosides modifying enzymes produced by the causative microorganisms. Huang et al. showed that gentamicin plus CZA typically had better *in vitro* activity against KPC-producing *K. pneumoniae* isolates with *aac (6′)-Ib*, while amikacin in combination with CZA exhibited more favorable activity against the isolates with *aac (6′)-Ib’* (31).

Given the limited evidence on CZA monotherapy vs. CZA-containing combination therapies, we definitely need further studies to address some important questions. First, pathogen-directed RCTs are urgently needed to fill this evidence gap considering the high-certainty evidence provided by RCTs. Second, large-scale observational studies should be conducted to understand efficacy and safety of CZA monotherapy and combination therapies in real life conditions and for some patients who are not eligible to be included in a RCT. In these observational studies, biases (e.g., selection bias, immortal time bias) and effects of relevant confounding factors should be mitigated using appropriate statistical methods. For this purpose, depending on the investigated outcomes, inverse probability treatment weighting using the propensity score, propensity score matching, multivariable logistic regression or Cox regression analyses can be performed (77). If there is a risk of immortal time bias in the survival analysis, time dependent Cox regression can be chosen to reduce this bias (78, 79). Third, genomic characterization of the causative bacteria should be undertaken to direct a precision medicine approach. Fourth, optimal dosing and infusion regimens of CZA and companion antibiotics and their treatment durations should be explored in future studies. Fifth, the impact of high-inoculum infections on the efficacy of CZA treatment should be investigated. Although *in vitro* studies demonstrated the resilience of CZA against high inoculum effect, the clinical activity of CZA monotherapy vs. combination therapies are not yet compared in high-inoculum infections such as osteomyelitis and endocarditis (80). Sixth, *in vitro* activity of CZA diminishes against MDR-PA isolates identified in sputum samples of cystic fibrosis patients compared to those isolated from other clinical samples (81). *P. aeruginosa* can form a biofilm and grow anaerobically in the cystic fibrosis patients’ lower respiratory tract (82). The biofilm structure enables *P. aeruginosa* strains to resist the actions of both antibiotics and the immune response (83). Considering the difficulties of treatment of these infections, the combination regimens including CZA and an *in vitro* active aminoglycoside or polymyxins may be more reasonable option than CZA monotherapy for the treatment of these patients. Future studies addressing this question are required to optimize treatment recommendations in this group. Lastly, clinical evidence is lacking on the efficacy and safety of CZA monotherapy vs. combination therapies for infections caused by pathogens with CZA MIC value very close to the susceptibility breakpoints.

## 5. Conclusion

Although *in vitro* studies have shown positive results with some CZA combination regimens, clinical studies have generally not found any clinical benefit with CZA combination therapies. Despite these findings, the potential benefit of CZA-containing combination therapies remains controversial for some specific patient groups. In addition, there is an unmet need for RCTs comparing CZA monotherapy and CZA-containing combination therapies.

## Data availability statement

The original contributions presented in the study are included in the article/Supplementary material, further inquiries can be directed to the corresponding author.

## Author contributions

AA: conceptualization, methodology, literature search, and drafting of the manuscript. YE: conceptualization, literature search, and editing and approval of manuscript. JH: literature search and editing and approval of manuscript. PH and DP: conceptualization, supervision, and editing and approval of manuscript. All authors contributed to the article and approved the submitted version.

## Conflict of interest

PH has received research grants from Merck Sharpe & Dohme (MSD), Sandoz and Shionogi outside of the submitted work; speakers fees from Pfizer, Sandoz, OpGen and Sumitomo and has served on advisory boards for Sandoz, OpGen and MSD. JH has received speaker fees from Pfizer, MSD, Angelini and Zambon, and has served on advisory boards for Menarini, Pfizer and TFF Pharmaceuticals. DP has received research grants from Shionogi, Pfizer, bioMerieux and Merck, and has served on advisory boards for Pfizer, Merck, Entasis, VenatoRx, Qpex and the AMR Action Fund.

The remaining authors declare that the research was conducted in the absence of any commercial or financial relationships that could be construed as a potential conflict of interest.

## Publisher’s note

All claims expressed in this article are solely those of the authors and do not necessarily represent those of their affiliated organizations, or those of the publisher, the editors and the reviewers. Any product that may be evaluated in this article, or claim that may be made by its manufacturer, is not guaranteed or endorsed by the publisher.

## Figures and Tables

**Table:** 

CRE	carbapenem-resistant *Enterobacterales*
MDR-PA	multidrug-resistant *Pseudomonas aeruginosa*
CZA	ceftazidime-avibactam
MBL	metallo-β-lactamase
AMR	Antimicrobial resistance
CDC	Centre for Disease Control and Prevention
DTR	difficult-to-treat resistant
ESBL	extended-spectrum β-lactamases
RCT	randomized clinical trial
PRISMA-ScR	Preferred Reporting Items for Systematic reviews and Meta-Analyses extension for Scoping Reviews
CFU	colony forming unit
AKI	acute kidney injury
CRKP	carbapenem-resistant *Klebsiella pneumoniae*
CRPA	carbapenem-resistant *P. aeruginosa*
ESCMID	European Society of Clinical Microbiology and Infectious Diseases
IDSA	Infectious Diseases Society of America
