# Supplementary material for: In vitro, in vivo and clinical studies comparing the efficacy of ceftazidime-avibactam monotherapy with ceftazidime-avibactam-containing combination regimens against carbapenem-resistant Enterobacterales and multidrug-resistant Pseudomonas aeruginosa isolates or infections: a scoping review
Source: Front Med (Lausanne). 2023 Sep 4;10:1249030. doi: 10.3389/fmed.2023.1249030 (PMC10506411; doi:10.3389/fmed.2023.1249030)
Supplement: Supplementary file 1 [file Table_1.DOCX]

Supplementary Material

**Information sources**

o Pubmed

o CENTRAL (the Cochrane Central Register of Controlled Trials)

o OvidMedline

o OvidCINAHL

- Embase

o Web of Science

o Scopus

o Clinicaltrial.gov

| **Table S1: Search syntax summary** | | | | |
| --- | --- | --- | --- | --- |
| **Database: Pubmed, search date: 25/10/2021** | | | | |
| PICO domain | | Free terms | Subject heading / clinical Queries | Query (total#) |
| Intervention | | ceftazidime/avibactam  ceftazidime-avibactam  avibactam  NXL104  AVE1330A | "avibactam, ceftazidime drug combination" [Supplementary concept] | (((ceftazidime) AND (avibactam)) OR ((((ceftazidime/avibactam) OR (ceftazidime-avibactam)) OR (NXL104)) OR (AVE1330A))) OR (("avibactam, ceftazidime drug combination" [Supplementary Concept])) |
| (("ceftazidime"[MeSH Terms] OR "ceftazidime"[All Fields] OR "ceftazidim"[All Fields]) AND ("avibactam"[Supplementary Concept] OR "avibactam"[All Fields])) OR ("avibactam ceftazidime drug combination"[Supplementary Concept] OR "avibactam ceftazidime drug combination"[All Fields] OR "ceftazidime avibactam"[All Fields] OR ("avibactam ceftazidime drug combination"[Supplementary Concept] OR "avibactam ceftazidime drug combination"[All Fields] OR "ceftazidime avibactam"[All Fields]) OR ("avibactam"[Supplementary Concept] OR "avibactam"[All Fields] OR "nxl104"[All Fields]) OR ("ave 1330a"[Supplementary Concept] OR "ave 1330a"[All Fields] OR "ave1330a"[All Fields])) OR "avibactam ceftazidime drug combination"[Supplementary Concept] | | | | |
| 1115 | | | | |
| **Database Scopus, Search date:25/10/2021** | | | | |
| Query | TITLE-ABS-KEY ( ( "ceftazidime/avibactam" OR "ceftazidime-avibactam" OR avibactam OR nxl104 OR ave1330a OR ( ceftazidime AND avibactam | | | |
| Total # | 1951 | | | |

| **Database: MEDLINE and CINAHL via EBSCOhost , Search date:25/10/2021** | |
| --- | --- |
| Query | SU ceftazidime/avibactam OR ceftazidime-avibactam OR NXL104 OR AVE1330A OR ceftazidime avibactam |
| Total # | 1118 |

| **Database: Web of Science, search date: 8/11/2021** | | | |
| --- | --- | --- | --- |
| PICO domain | Free terms | Subject heading / clinical Queries | Query (total#) |
| Intervention | ceftazidime/avibactam ceftazidime-avibactam  avibactam  NXL104  AVE1330A |  | TS=(ceftazidime/avibactam OR ceftazidime-avibactam OR avibactam OR NXL104 OR AVE1330A) OR TI=(ceftazidime/avibactam OR ceftazidime-avibactam OR avibactam OR NXL104 OR AVE1330A) |
| Final Query  TS=(ceftazidime/avibactam OR ceftazidime-avibactam OR avibactam OR nxl104 OR ae1330a) OR TI=(ceftazidime/avibactam OR ceftazidime-avibactam OR avibactam OR nxl104 OR ae1330a) OR AB=(ceftazidime/avibactam OR ceftazidime-avibactam OR avibactam OR nxl104 OR ae1330a) | | | |
| Total 303 | | | |

| **Database: Cochrane, Search date. Date Run:25/10/2021** | |
| --- | --- |
| Query | Title Abstract Keyword  ceftazidime-avibactam  OR  Title Abstract Keyword  "ceftazidime avibactam"  OR  Title Abstract Keyword  NXL104  OR  Title Abstract Keyword  AVE1330A |
| Total # | 88 (trials) |

| **Database: Embase, search date: 08/11/2021** | | | |
| --- | --- | --- | --- |
| Top 5 Therapy questions | Free terms | Subject heading / clinical Queries | Query (total#) |
| Intervention | ceftazidime/avibactam (no findings)  ceftazidime-avibactam  avibactam  NXL104  AVE1330A |  | ('ceftazidime avibactam'/exp OR 'ceftazidime avibactam') OR ('avibactam'/exp OR avibactam) OR ('nxl104'/exp OR nxl104) OR ('ave1330a'/exp OR ave1330a) |
| Final Query and total # P+I+C (without publication filter  'ceftazidime avibactam'/exp OR 'ceftazidime avibactam' OR 'avibactam' OR 'avibactam'/exp OR avibactam OR 'nxl104' OR 'nxl104'/exp OR nxl104 OR 'ave1330a' OR 'ave1330a'/exp OR ave1330a | | | |
| Total 2384 | | | |

| **Database name** | **Number of articles found** | **Number of duplicates** | **Number of records screened** |
| --- | --- | --- | --- |
| Medline and CINAHL | 1117 | 673 | 444 |
| Cochrane | 88 | 34 | 54 |
| Embase | 2384 | 1922 | 462 |
| Pubmed | 1115 | 975 | 140 |
| Scopus | 1951 | 1354 | 597 |
| Web of Science | 303 | 110 | 193 |
| Total | 6958 | 5068 | 1890 |

| **Excluded on bases of title and abstracts** | |
| --- | --- |
| total | 1440 |

| **Excluded on bases of full text papers (Reason)** | |
| --- | --- |
| Out of Scope of SR | 254 |
| Review | 36 |
| Abstract only | 35 |
| Protocol | 24 |
| Incomplete data | 19 |
| Commentary/letter/opinion paper | 10 |
| Children | 7 |
| Duplication | 6 |
| Non-English | 6 |
| Guideline | 3 |
| Only one isolate tested (in vitro study) | 1 |
| total | 401 |
